# Supplementary material for: “What If I Die and No One Notices?” A Qualitative Study Exploring How Living Alone and in Poverty Impacts the Health and Well-Being of Older People in Hong Kong
Source: Int J Environ Res Public Health. 2022 Nov 28;19(23):15856. doi: 10.3390/ijerph192315856 (PMC9739217; doi:10.3390/ijerph192315856)
Supplement: Supplementary file 1 [file ijerph-19-15856-s001.zip › ijerph-2034586-supplementary.pdf]

Supplementary Material

Research Design

This study employed a qualitative design using life history interviews [1,2]. Life history interviews lasting from 1 to 1.5 hours were conducted with each participant, for a total of 47 interviews (see Appendix A for the interview guide). Life history interviews differ from more generic, semi-structured interviews, in that they are longer in length (as the aim is to elicit a more in-depth and historical understanding of the phenomenon of interest), and they adopt a chronological approach to developing a narrative [2]. In the absence of longitudinal panel study data that follow the same households over long periods of time, life history interviews provide a promising alternative, as they are a less resource-intense method for collecting longitudinal data. Furthermore, this study sought to prioritize and engage with the experiences and perspectives of older adults who were living in poverty, because their voices are often left out of scholarly and policy discourse [3]. Qualitative inquiry is a natural fit for facilitating the voices of this marginalized group, because it focuses on creating knowledge concerning participants' experiences with the phenomenon of interest, primarily through the lens of the participant rather than that of the researcher [4].

Target Population, Sample, and Recruitment

For the study's sample, we used purposive sampling [5] to select as participants older adults who were living alone. The inclusion criteria for selecting respondents were that they be: i) 65 years of age or older, ii) living alone, iii) income-poor, on the basis of the Hong Kong official poverty threshold, iv) living in Hong Kong, for any length of residence, and v) not residing in owner-occupied housing (and not owning a home). The last criterion recognizes a specific subcohort of "asset-rich and income-poor" older adults who, although considered poor by official poverty thresholds, are in fact not poor, because "owner-occupied housing is an important asset in the investment portfolio of many families, therefore its value itself can serve as a reference basis of the value of assets owned by households" [6]. Also, other research suggests that older adults who own a home are less likely to be poor [7]. Individuals who have cognitive or memory impairments and health issues that may prevent them from recalling past events and/or challenge their ability to sit through a long interview (1.5 to 2 hours) also were excluded.

Regarding sample size, an initial quota of 20 females living alone (n = 20) and 20 males living alone (n = 20) was determined. However, it was challenging to recruit males living alone, so the final sample set was 37 females living alone (n = 37) and 10 males living alone (n = 10). A local NGO that provided existing services for older adults who were living alone and in poverty in all of the 18 districts in Hong Kong was engaged as a partner in the study and helped with recruitment. To recruit participants, an information sheet (providing a brief plain-language summary of the study, and including specifically the inclusion criteria) and the informed consent form, was provided to the various district offices. If an office had a participant who was interested, they asked the person's permission to provide their contact information to the research team. The research team then contacted the participant to arrange an interview.

Data Analysis

The interviews were audio recorded with the permission of the participants, and then transcribed. The data collected for analysis for the older adults who were living alone comprised 47 transcripts. To manage the large volume of data, analysis was conducted continuously and recursively alongside data collection (see Table 1 for details of the data analysis procedure). This study employed the six-stage method of thematic analysis (TA), with a hybrid approach to coding, whereby possible codes were derived from the literature [4,8]. NVivo, a qualitative analysis software tool, supported the data analysis process. Table 1 describes the data analysis procedure. Both authors were involved in data analysis.

Table S1. Data Analysis Procedures

Stage	Protocols for each stage of the study
1. Organization and preparation of the transcript for analysis [4]	• Identification and arrangement of the transcript, on the basis of the source of the information/case (e.g., female living alone or male living alone, and which interview was used: 1, 2, or 3)<br>• Division of each interview transcript into sections according to whether it corresponded to chronological stages in life (childhood, young adulthood, adulthood, older adulthood)
2. Development of the initial code book [8]	• Adoption of a hybrid approach to coding, because the literature contained insufficient evidence suggesting possible codes to explore (e.g., social support, housing insecurity, employment)<br>• Extraction of segments when coding a transcript. A segment entailed either a phrase, a sentence, a section of a paragraph, or a paragraph, but was not a single word, nor was it longer than a paragraph.<br>• Meeting of the analysts, when five transcripts were coded independently by the first author and the second author, to discuss and create the initial code book. The book had three columns: name of code, definition of code, and specific instances (e.g., line number of transcript) where the code could be found [4]
3. Revision of the code book	• Four subsequent meetings after the initial development of the code book to discuss new codes, resolve disagreements, and revise the code book accordingly.<br>• Each meeting was held after a new set of transcripts (e.g., 5-10) were coded independently by an analyst.
4. Generation of descriptions of the contextual background and the potential themes for analysis, using the codes [8]	• Generation of a description of the contextual background (such as sociodemographic information) of each case, using the codes.<br>• Generation of a small number of potential themes for analysis *within* each case, and then *across* the two cases, again using the codes.<br>• Identification of codes obtaining information that contradicted/diverged from the potential themes, as a validity strategy (described in detail in stage 5).
5. Identification and revision of themes [8]	• Identification of key themes, selecting those that occurred with at least half of the participants (n = 24)<br>o For a theme to occur *within* a case, there had to be evidence from at least half of the cases (e.g., n = 19 for females living alone and n = 5 for males living alone).<br>o Identification of a unique theme was based not on the number of occurrences but rather on whether it was counter-evidence against any of the key themes, and/or provided an important insight into the research aim
6. Definition and labeling of the final themes [8]	• Formal definition and labeling of each potential theme as they would later be expressed in the research dissemination products (e.g., manuscript, reports)<br>• Testing of the clarity and conciseness of the themes. The team at this stage described the themes in 1 to 2 sentences. If we were unable to do this, then further revisions and discussion of the theme were made

	(e.g., can this theme be broken down further into two or more clear themes?)

Reliability and Validity Procedures

Life history interviews have the limitation of relying on recall from earlier periods and may be incomplete or skewed [9]. As a reliability strategy to address this limitation, we invited respondents to report only events that had made a significant impact on their well-being and to offer evidence to indicate that impact (e.g., death of a spouse, event leading to the need to re-enter the labor market and/or sell substantial household assets, etc.). This aligned with Krishna's [9] directive that "seeking recall data in terms of these clear, conspicuous, and sizeable referents . . . adds reliability to recall" (p. 194).

In this study, we also used reliability strategies for data analysis, including conducting five regular and documented meetings between the first and second authors to discuss and resolve agreements, disagreements, and emerging issues throughout the analytic process. To increase validity, the findings were conveyed in a descriptively rich way to provide the reader with a vicarious experience and with sufficient details of both the setting/context and the perspectives of the themes [4]. In addition to presenting the major themes, any counter-evidence collected (in stage 4 of the TA procedure) was presented with full transparency (e.g., Theme 4 in the manuscript: "Most of us like to be alone," was in contrast to Theme 1: "Social isolation and loneliness: What if I die and no one notices?"), in recognition of the fact that "real life is composed of different perspectives that do not always coalesce, [so] discussing contrary information adds to the credibility of the account" [4].

Appendix References

1. Davis, P. Poverty in time: Exploring poverty dynamics from life history interviews in Bangladesh. In T. Addison, D. Hulme, & R. Kanbur (Eds.), Poverty dynamics: Interdisciplinary perspectives (pp. 154–182). **2009**. Oxford University Press. Oxford, England.
2. Jessee, E. The life history interview. In P. Liamputtong (Ed.), Handbook of research methods in health social sciences (pp. 1–17). **2018**. Springer. New York.
3. Kwan, C.; Walsh, C. Old age poverty: A scoping review of the literature. *Cogent Social Sciences*, **2018**, 4(1), 1-21. https://doi.org/10.1080/23311886.2018.1478479
4. Creswell, J. W. Research design: Qualitative, quantitative, and mixed methods approaches (3rd ed.). **2009**. SAGE. Thousand Oaks, CA.
5. Trotter, R.T. Qualitative research sample design and sample size: Resolving and unresolved issues and inferential imperatives. *Preventive Medicine*, **2012**, 55(5), 398–400. https://doi.org/10.1016/j.ypmed.2012.07.003
6. Government of the Hong Kong Special Administrative Region. *Hong Kong poverty situation report 2017*, **2018**. Retrieved from https://www.povertyrelief.gov.hk/eng/pdf/Hong_Kong_Poverty_Situation_Report_2017(2018.11.19).pdf
7. Chou, K.; Lee, S. Superimpose material deprivation study on poverty old age people in Hong Kong Study. *Soc. Indic. Res.* **2018**, 139, 1015–1036.
8. Braun, V.; Clarke, V. Using thematic analysis in psychology. *Qualitative Research in Psychology*, **2006**, 3(2), 77-101. https://doi.org/10.1191/1478088706qp063oa
9. Krishna, A. Subjective assessments, participatory methods, and poverty dynamics: The stages of progress method. In T. Addison, D. Hulme, & R. Kanbur (Eds.), Poverty dynamics: Interdisciplinary perspectives (pp. 183–204). **2009**, Oxford University Press. Oxford, England.
